# Serial Serum Creatine Phosphokinase and Lactate Dehydrogenase Levels as Biomarkers of Severity and Intermediate Syndrome in Organophosphorus Compound Poisoning: A Prospective Observational Study

**DOI:** 10.7759/cureus.115627

**Published:** 2026-09-01

**Authors:** Pulkit Singhal, Amit Kumar Sagar, Surendra Singh Bhakal, Ramesh Chandra, Tejpal Yadav, Jawar Solanki, Hitesh Purohit, Dinesh Jandu, Arpit Mittal, Yash Bothra

**Affiliations:** 1 General Medicine, Dr. Sampurnanand Medical College, Jodhpur, IND

**Keywords:** biomarkers, creatine phosphokinase, intermediate syndrome, lactate dehydrogenase, organophosphorus poisoning, peradeniya organophosphorus poisoning, poisoning severity

## Abstract

Background

Organophosphorus compound poisoning remains an important cause of morbidity and mortality in agrarian regions. Early identification of patients at risk of worsening toxicity and intermediate syndrome remains challenging. Serum creatine phosphokinase and lactate dehydrogenase may reflect neuromuscular and tissue injury and may therefore assist clinical risk stratification.

Methods

This prospective observational study included 80 adult patients with mild-to-moderate organophosphorus compound poisoning admitted to a tertiary care teaching hospital between March 2025 and June 2025. Poisoning severity was assessed using a modified Peradeniya Organophosphorus Poisoning (POP)-based clinical score. Serum creatine phosphokinase and lactate dehydrogenase levels were measured at admission, 48 hours, and 96 hours. Associations with poisoning severity and intermediate syndrome were evaluated using the Mann-Whitney U test, correlations with the severity score using Pearson correlation, and discriminative performance using receiver operating characteristic curve analysis.

Results

The mean age was 26.69 ± 8.70 years, 43 patients (53.8%) were male, and accidental exposure was the predominant mode of poisoning, observed in 60 patients (75.0%). Intermediate syndrome developed in eight patients (10.0%), and three patients (3.8%) died. Serum creatine phosphokinase and lactate dehydrogenase levels were significantly higher in moderate poisoning than in mild poisoning at admission, 48 hours, and 96 hours (all p < 0.001). Patients who developed intermediate syndrome had higher biomarker levels throughout hospitalization, including creatine phosphokinase at admission (1281.88 ± 631.83 vs. 434.32 ± 304.95 IU/L; p = 0.001) and lactate dehydrogenase at 48 hours (1516.88 ± 777.95 vs. 528.47 ± 303.78 IU/L; p = 0.001). Biomarker levels correlated positively with the severity score, with the strongest correlations observed for lactate dehydrogenase at admission (r = 0.852, p < 0.001) and creatine phosphokinase at 96 hours (r = 0.687, p < 0.001). Receiver operating characteristic analysis showed excellent discrimination for moderate poisoning and good discrimination for intermediate syndrome.

Conclusions

Serial serum creatine phosphokinase and lactate dehydrogenase levels were significantly associated with poisoning severity and intermediate syndrome in mild-to-moderate organophosphorus compound poisoning. These inexpensive and widely available biomarkers may be useful adjuncts to clinical assessment, although they should not replace careful monitoring and validated severity assessment. Mortality-related findings require cautious interpretation because only three deaths occurred.

## Introduction

Organophosphorus compound poisoning remains a major public health problem in developing countries, particularly in agrarian regions where pesticide availability and occupational exposure are common [1]. Acute poisoning is associated with significant morbidity and mortality, with severe complications including respiratory failure, intermediate syndrome, prolonged hospitalization, and death [1].

Organophosphate toxicity results from inhibition of acetylcholinesterase, causing excessive accumulation of acetylcholine at muscarinic, nicotinic, and central nervous system synapses [2,3]. Although acute cholinergic manifestations may improve after atropinization and supportive care, delayed neuromuscular complications can occur. Intermediate syndrome usually develops 24 to 96 hours after poisoning and is characterized by weakness of proximal limb muscles, neck flexors, respiratory muscles, and cranial nerve-innervated muscles [4,5]. Timely recognition is important because respiratory muscle involvement may require ventilatory support. Despite standard treatment with atropine and oximes, clinical progression remains variable, and early identification of patients at risk of deterioration continues to be challenging [6].

Clinical severity is commonly assessed using bedside clinical evaluation and scoring systems such as the Peradeniya Organophosphorus Poisoning (POP) scale [7]. Serum cholinesterase estimation is frequently used in organophosphorus poisoning, but its correlation with clinical severity and delayed complications is inconsistent [3,8]. This has encouraged evaluation of alternative biochemical markers that are inexpensive, rapid, and widely available.

Neuromuscular injury and muscle fiber damage secondary to cholinergic overstimulation may elevate serum creatine phosphokinase (CPK) and lactate dehydrogenase (LDH) levels [9-11]. Prior studies have reported associations between elevated serum CPK levels, poisoning severity, intermediate syndrome, and adverse outcomes [9-12]. However, evidence regarding serial measurement of both CPK and LDH remains limited. The present study evaluated the association of serial serum CPK and LDH levels with poisoning severity, intermediate syndrome, and clinical outcomes in patients with organophosphorus compound poisoning.

## Materials and methods

Study design and setting

This prospective observational study was conducted in the Department of Medicine at Dr. Sampurnanand Medical College, including Mathuradas Mathur Hospital and Mahatma Gandhi Hospital, Jodhpur, Rajasthan, India.

Study duration

The study was conducted between March 2025 and June 2025 after obtaining approval from the Institutional Ethics Committee.

Study population

Adult patients presenting to the emergency departments with acute organophosphorus compound poisoning were screened for eligibility. Patients aged 18 years or older, of either sex, who presented within six hours of confirmed organophosphorus compound exposure and had mild-to-moderate poisoning severity according to the modified POP score were included. Patients with clinical features suggestive of organophosphorus poisoning, including miosis, salivation, lacrimation, diarrhea, muscle fasciculations, and related cholinergic manifestations, were considered eligible.

Exclusion criteria

Patients with severe poisoning at presentation, seizures, respiratory failure requiring immediate mechanical ventilation, Glasgow Coma Scale score below 8, chronic kidney disease, acute kidney injury, chronic liver disease, acute hepatic dysfunction, chronic alcohol use disorder, or any other condition known to independently alter serum CPK or LDH levels were excluded.

Sample size and sampling technique

Consecutive eligible patients fulfilling the inclusion criteria during the study period were enrolled using a non-random sampling technique. The sample size was calculated for comparison of mean serum CPK levels between two independent groups using assumptions derived from a previously published study by Nimsarkar et al. [13]. Using a two-sided alpha error of 0.05, study power of 90%, Zα/2 of 1.96, Zβ of 1.28, and the pooled variance derived from these published values, the minimum required sample size was calculated to be 18 participants in each group. To improve precision and maintain equal group representation, 80 patients were included, comprising 40 patients with mild poisoning and 40 patients with moderate poisoning.

Clinical severity assessment

Severity was assessed at admission using a modified clinical scoring system based on the POP scale. The original POP scale was developed by Senanayake et al. and was used as the supporting clinical severity framework in the present study [7]. The scoring system used in the present study incorporated seven clinical parameters: consciousness (0-3), pupil size (0-2), respiratory rate (0-2), muscle strength (0-3), fasciculations (0-1), tachycardia (0-3), and systolic blood pressure (0-3), giving a total possible score of 0-17. Based on the study protocol, scores of 0-5 were classified as mild, 6-11 as moderate, and 12-17 as severe poisoning. Only patients classified as having mild or moderate poisoning were included in the present analysis.

Data collection and laboratory assessment

After written informed consent was obtained, demographic details, exposure history, clinical findings, severity score, treatment details, and outcomes were recorded using a structured proforma. Serum CPK and LDH levels were measured at admission, 48 hours, and 96 hours using the DiaSys SYS 1000 clinical chemistry analyzer (DiaSys Diagnostics India Pvt. Ltd., Navi Mumbai, India). The laboratory reference ranges were 24-195 IU/L for serum CPK and 140-280 IU/L for serum LDH. Additional laboratory investigations included complete blood count, renal function tests, liver function tests, and serum electrolytes.

Definition and monitoring of intermediate syndrome

Patients were monitored throughout hospitalization for intermediate syndrome, respiratory distress, need for ventilatory support, atropine requirement, duration of hospitalization, and final outcome. Intermediate syndrome was defined clinically as delayed onset of weakness involving proximal limb muscles, neck flexors, cranial nerve-innervated muscles, or respiratory muscles after improvement of the acute cholinergic phase, typically within 24 to 96 hours after poisoning [4,5].

Treatment protocol

All patients received standard treatment according to institutional protocol, including atropine therapy, pralidoxime administration, and supportive care. Mechanical ventilation was provided when clinically indicated.

Outcome measures

The primary outcome measures were the association of serial serum CPK and LDH levels with poisoning severity and development of intermediate syndrome. Secondary outcome measures included their association with mortality, duration of hospitalization, and receiver operating characteristic (ROC) curve performance.

Statistical analysis

Statistical analysis was performed using IBM SPSS Statistics version 23.0 (IBM Corp., Armonk, NY). Continuous variables were assessed for normality using the Shapiro-Wilk test and were expressed as mean ± standard deviation. Between-group comparisons of serial CPK and LDH levels were performed using the Mann-Whitney U test. Categorical variables were expressed as n (%) and compared using the chi-square test or Fisher’s exact test, as appropriate according to expected cell counts. Correlations between POP-based severity scores and serial serum CPK and LDH levels were assessed using Pearson’s correlation coefficient. ROC curve analysis was performed to assess the discriminatory performance of serial CPK and LDH levels for moderate poisoning severity, intermediate syndrome, and mortality. As only three deaths occurred, mortality-related ROC findings were interpreted as exploratory. A p-value <0.05 was considered statistically significant.

Ethical considerations

Ethical approval was obtained from the Institutional Ethics Committee, Dr. Sampurnanand Medical College, Jodhpur, Rajasthan, India, before commencement of the study (Certificate Reference Number: SNMC/IEC/2025/Plan/1057; dated: 27.03.2025). Written informed consent was obtained from all participants or their legally acceptable representatives, and confidentiality of participant data was maintained throughout the study.

## Results

Baseline demographic and clinical characteristics

A total of 80 patients with organophosphorus compound poisoning were included. The mean age was 26.69 ± 8.70 years, and 43 patients (53.8%) were male. Accidental exposure was the predominant mode of poisoning, occurring in 60 patients (75.0%). Chlorpyrifos was the most common recorded exposure, accounting for 68 cases (85.0%). Mild and moderate poisoning groups were equally represented, with 40 patients (50.0%) in each group. Intermediate syndrome developed in eight patients (10.0%), and three patients (3.8%) died during hospitalization. The mean duration of hospital stay was longer in patients with moderate poisoning than in those with mild poisoning (11.42 ± 2.26 vs. 7.58 ± 1.34 days) and was also longer in patients who developed intermediate syndrome than in those who did not (14.00 ± 1.31 vs. 9.00 ± 2.30 days) (Table 1).

**Table 1:** Baseline clinical characteristics and outcomes of the study population. Data are presented as N (%) for categorical variables and mean ± SD for continuous variables. SD, standard deviation; IMS, intermediate syndrome.

Parameter	Category	N (%) or mean ± SD
Age (years)	Mean ± SD	26.69 ± 8.70
Age distribution	<=25 years	41 (51.2%)
	26-35 years	32 (40.0%)
	36-45 years	3 (3.8%)
	46-55 years	2 (2.5%)
	>55 years	2 (2.5%)
Sex	Male	43 (53.8%)
	Female	37 (46.2%)
Mode of exposure	Accidental	60 (75.0%)
	Intentional	20 (25.0%)
Most common recorded agent	Chlorpyrifos	68 (85.0%)
	Other recorded pesticide agents	12 (15.0%)
Poisoning severity	Mild	40 (50.0%)
	Moderate	40 (50.0%)
Intermediate syndrome	Yes	8 (10.0%)
	No	72 (90.0%)
Outcome	Survival	77 (96.2%)
	Mortality	3 (3.8%)
Duration of hospital stay (days)	Mean ± SD	9.50 ± 2.68
Duration of hospital stay by severity (days)	Mild poisoning	7.58 ± 1.34
	Moderate poisoning	11.42 ± 2.26
Duration of hospital stay by IMS status (days)	IMS absent	9.00 ± 2.30
	IMS present	14.00 ± 1.31

Serial serum CPK levels

Serum CPK levels demonstrated an early rise during the first 48 hours, followed by a decline at 96 hours. Mean CPK levels were 519.08 ± 429.44 IU/L at admission, 599.60 ± 449.79 IU/L at 48 hours, and 353.35 ± 224.82 IU/L at 96 hours. Patients with moderate poisoning had significantly higher CPK levels than patients with mild poisoning at all three time points (all p < 0.001). Patients who developed intermediate syndrome also had higher CPK levels throughout hospitalization (Figure 1 and Tables 2, 3).

**Figure 1:**
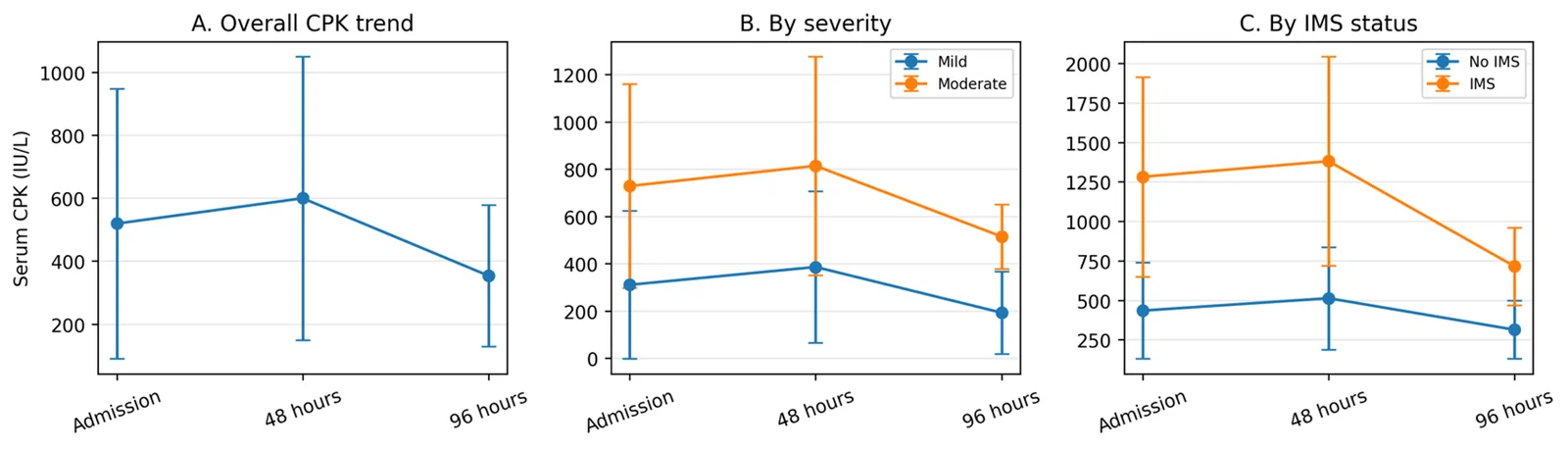
Serial serum creatine phosphokinase levels in organophosphorus compound poisoning. Panel A shows the overall temporal trend in serum creatine phosphokinase (CPK) levels at admission, 48 hours, and 96 hours. Panel B compares CPK levels between patients with mild and moderate poisoning. Panel C compares CPK levels between patients with and without intermediate syndrome. Data are presented as mean ± standard deviation (SD). CPK, creatine phosphokinase; IMS, intermediate syndrome.

**Table 2:** Serial biomarker levels according to poisoning severity. Values are presented as mean ± SD. Between-group comparisons were performed using the Mann-Whitney U test, with the corresponding U statistic reported in the table. Statistical significance was defined as p < 0.05. CPK, creatine phosphokinase; LDH, lactate dehydrogenase; SD, standard deviation.

Parameter	Mild (n = 40), mean ± SD	Moderate (n = 40), mean ± SD	Total (N = 80), mean ± SD	Test statistic (Mann-Whitney U)	p-value
CPK admission (IU/L)	310.00 ± 313.10	728.15 ± 431.13	519.08 ± 429.44	72.0	<0.001
CPK 48 h (IU/L)	385.43 ± 319.98	813.78 ± 461.89	599.60 ± 449.79	71.0	<0.001
CPK 96 h (IU/L)	192.15 ± 174.81	514.55 ± 136.11	353.35 ± 224.82	77.0	<0.001
LDH admission (IU/L)	263.32 ± 75.32	587.23 ± 143.25	425.28 ± 198.72	35.0	<0.001
LDH 48 h (IU/L)	402.78 ± 341.79	851.85 ± 486.57	627.31 ± 474.98	79.0	<0.001
LDH 96 h (IU/L)	184.30 ± 173.03	489.28 ± 141.12	336.79 ± 219.45	78.0	<0.001

**Table 3:** Serial biomarker levels according to intermediate syndrome status. Values are presented as mean ± standard deviation (SD). Between-group comparisons were performed using the Mann-Whitney U test, with the corresponding U statistic reported in the table. A p-value < 0.05 was considered statistically significant. CPK, creatine phosphokinase; LDH, lactate dehydrogenase; SD, standard deviation; IMS, intermediate syndrome.

Parameter	No IMS (n = 72), mean ± SD	IMS (n = 8), mean ± SD	Total (N = 80), mean ± SD	Test statistic (Mann-Whitney U)	p-value
CPK admission (IU/L)	434.32 ± 304.95	1281.88 ± 631.83	519.08 ± 429.44	82.0	0.001
CPK 48 h (IU/L)	512.81 ± 324.83	1380.75 ± 661.96	599.60 ± 449.79	100.5	0.003
CPK 96 h (IU/L)	313.14 ± 183.98	715.25 ± 246.60	353.35 ± 224.82	79.0	0.001
LDH admission (IU/L)	407.78 ± 188.72	582.75 ± 230.11	425.28 ± 198.72	160.0	0.040
LDH 48 h (IU/L)	528.47 ± 303.78	1516.88 ± 777.95	627.31 ± 474.98	79.5	0.001
LDH 96 h (IU/L)	298.64 ± 179.99	680.13 ± 255.38	336.79 ± 219.45	88.0	0.001

Serial serum LDH levels

Serum LDH levels showed a similar temporal pattern, increasing from 425.28 ± 198.72 IU/L at admission to 627.31 ± 474.98 IU/L at 48 hours, followed by a decline to 336.79 ± 219.45 IU/L at 96 hours. Patients with moderate poisoning had significantly higher LDH levels than patients with mild poisoning at all measured time points (all p < 0.001). LDH levels were also higher among patients who developed intermediate syndrome, including at admission and during follow-up (Figure 2 and Tables 2, 3).

**Figure 2:**
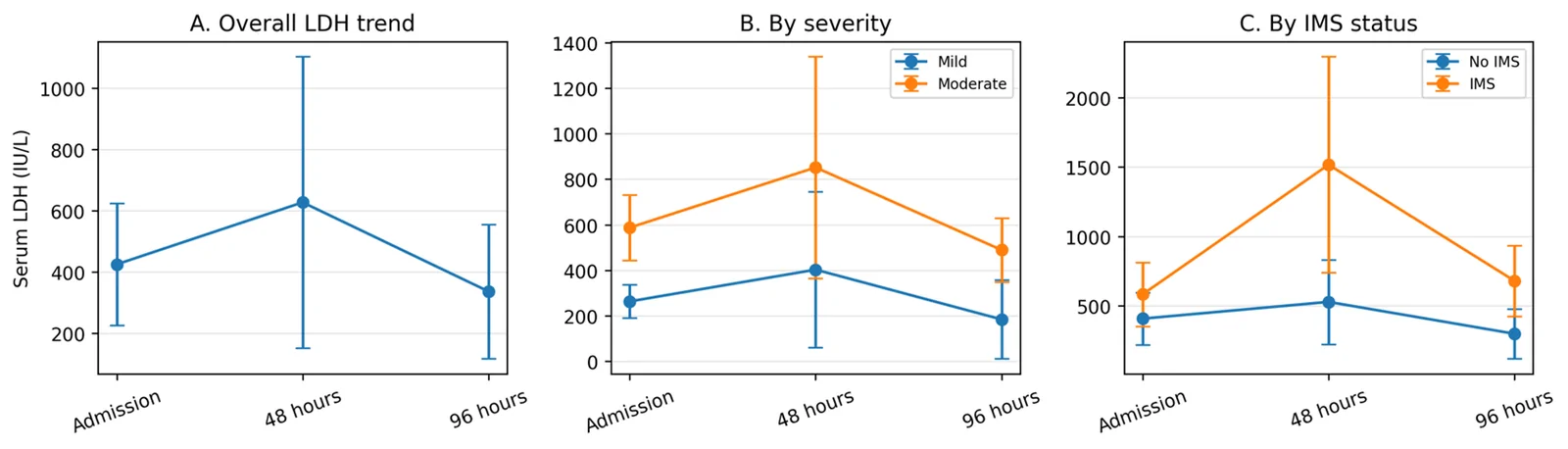
Serial serum lactate dehydrogenase levels in organophosphorus compound poisoning. Panel A shows the overall temporal trend in serum lactate dehydrogenase (LDH) levels at admission, 48 hours, and 96 hours. Panel B compares LDH levels between patients with mild and moderate poisoning. Panel C compares LDH levels between patients with and without intermediate syndrome. Data are presented as mean ± standard deviation (SD). LDH, lactate dehydrogenase; IMS, intermediate syndrome; SD, standard deviation.

Correlation of severity score with serum biomarkers

The severity score showed significant positive correlations with serial serum CPK and LDH levels at admission, 48 hours, and 96 hours. The strongest correlations were observed for LDH at admission (r = 0.852, p < 0.001) and CPK at 96 hours (r = 0.687, p < 0.001) (Table 4).

**Table 4:** Correlation of severity score with serial serum creatine phosphokinase and lactate dehydrogenase levels. Values are presented as Pearson correlation coefficients (r) with corresponding p-values. A p-value < 0.05 was considered statistically significant. CPK, creatine phosphokinase; LDH, lactate dehydrogenase.

Parameter	Correlation coefficient (r)	p-value
CPK admission (IU/L)	0.491	<0.001
CPK 48 h (IU/L)	0.480	<0.001
CPK 96 h (IU/L)	0.687	<0.001
LDH admission (IU/L)	0.852	<0.001
LDH 48 h (IU/L)	0.468	<0.001
LDH 96 h (IU/L)	0.671	<0.001

Receiver operating characteristic curve analysis

ROC curve analysis showed excellent discrimination of moderate poisoning by all serial biomarker measurements. LDH at admission showed the highest area under the curve (AUC) for moderate poisoning (AUC = 0.978; 95% CI, 0.955-1.000; p < 0.001). For intermediate syndrome, CPK at 96 hours (AUC = 0.863; 95% CI, 0.732-0.994; p = 0.001) and LDH at 48 hours (AUC = 0.862; 95% CI, 0.720-1.000; p = 0.001) showed the highest discrimination. Mortality analysis was exploratory because only three patients (3.8%) died; LDH at 48 hours had the highest mortality AUC (0.853; 95% CI, 0.641-1.000; p = 0.039) (Table 5 and Figures 3-5).

**Table 5:** Receiver operating characteristic curve analysis of serial biomarkers. AUC values were calculated using nonparametric ROC analysis, with the null hypothesis that the true AUC equals 0.5. Values are presented as AUC, standard error, 95% confidence interval, and p-value. A p-value < 0.05 was considered statistically significant. Mortality-related ROC findings should be interpreted cautiously because only three patients (3.8%) died. ROC, receiver operating characteristic; AUC, area under the curve; CI, confidence interval; CPK, creatine phosphokinase; LDH, lactate dehydrogenase; IMS, intermediate syndrome.

Outcome	Test variable	AUC	Standard error	95% CI	p-value
Moderate poisoning	CPK admission	0.955	0.031	0.894-1.000	<0.001
	CPK 48 h	0.956	0.031	0.895-1.000	<0.001
	CPK 96 h	0.952	0.033	0.887-1.000	<0.001
	LDH admission	0.978	0.012	0.955-1.000	<0.001
	LDH 48 h	0.951	0.032	0.888-1.000	<0.001
	LDH 96 h	0.951	0.034	0.885-1.000	<0.001
Intermediate syndrome	CPK admission	0.858	0.066	0.729-0.986	0.001
	CPK 48 h	0.826	0.075	0.679-0.972	0.003
	CPK 96 h	0.863	0.067	0.732-0.994	0.001
	LDH admission	0.722	0.083	0.560-0.884	0.040
	LDH 48 h	0.862	0.072	0.720-1.000	0.001
	LDH 96 h	0.847	0.072	0.707-0.988	0.001
Mortality	CPK admission	0.818	0.131	0.562-1.000	0.063
	CPK 48 h	0.823	0.127	0.573-1.000	0.059
	CPK 96 h	0.807	0.132	0.549-1.000	0.072
	LDH admission	0.823	0.127	0.574-1.000	0.059
	LDH 48 h	0.853	0.108	0.641-1.000	0.039
	LDH 96 h	0.797	0.128	0.546-1.000	0.083

**Figure 3:**
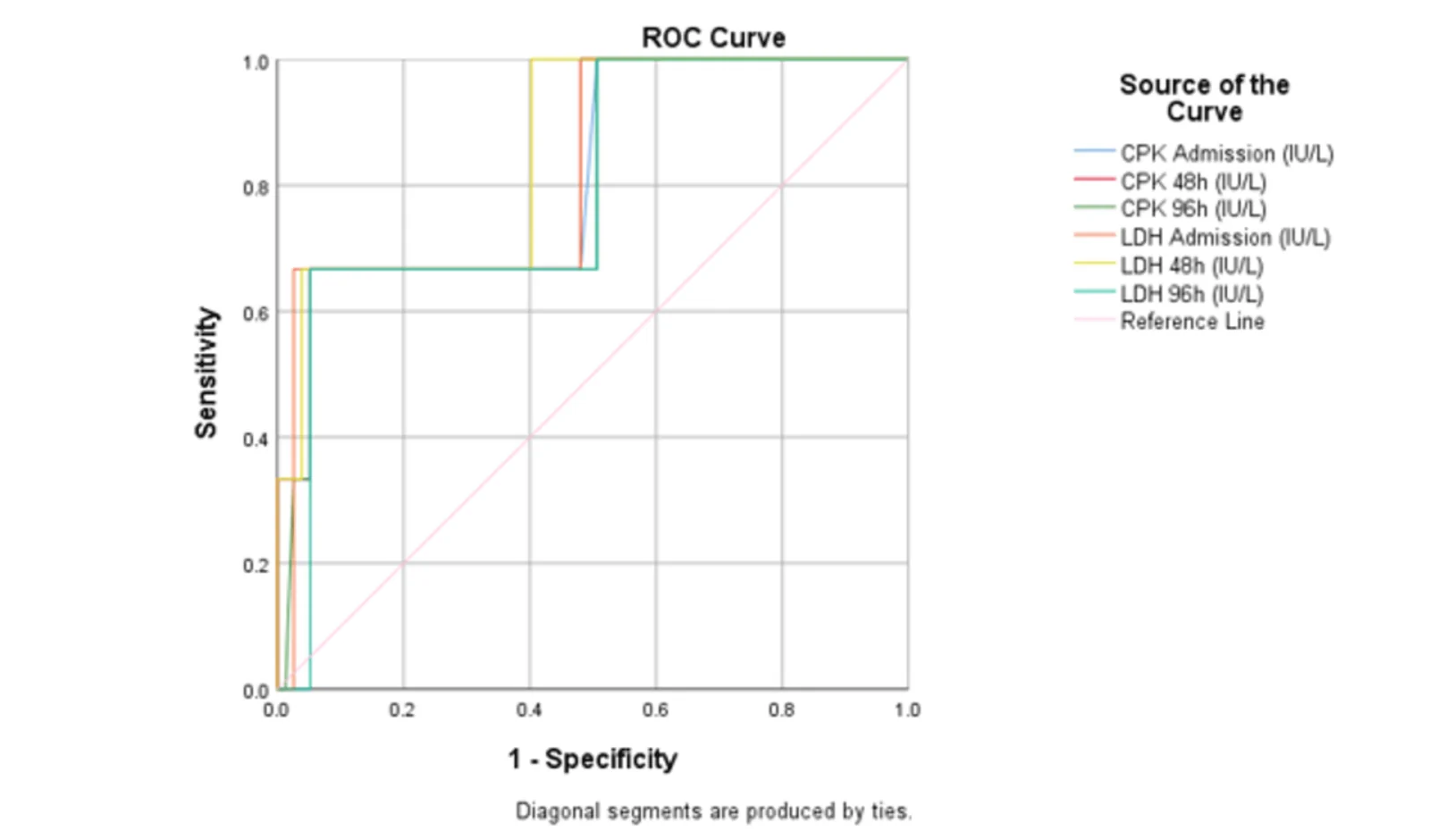
Receiver operating characteristic curves for mortality. Curves are shown for serial CPK and LDH levels at admission, 48 hours, and 96 hours, using mortality as the positive actual state. Sensitivity is plotted against 1 − specificity. Discriminative performance is expressed as the area under the receiver operating characteristic curve (AUC), with corresponding 95% confidence intervals and p-values reported in Table 5. Statistical significance was defined as p < 0.05. This analysis should be interpreted as exploratory because only three patients (3.8%) died. CPK, creatine phosphokinase; LDH, lactate dehydrogenase; ROC, receiver operating characteristic; AUC, area under the curve.

**Figure 4:**
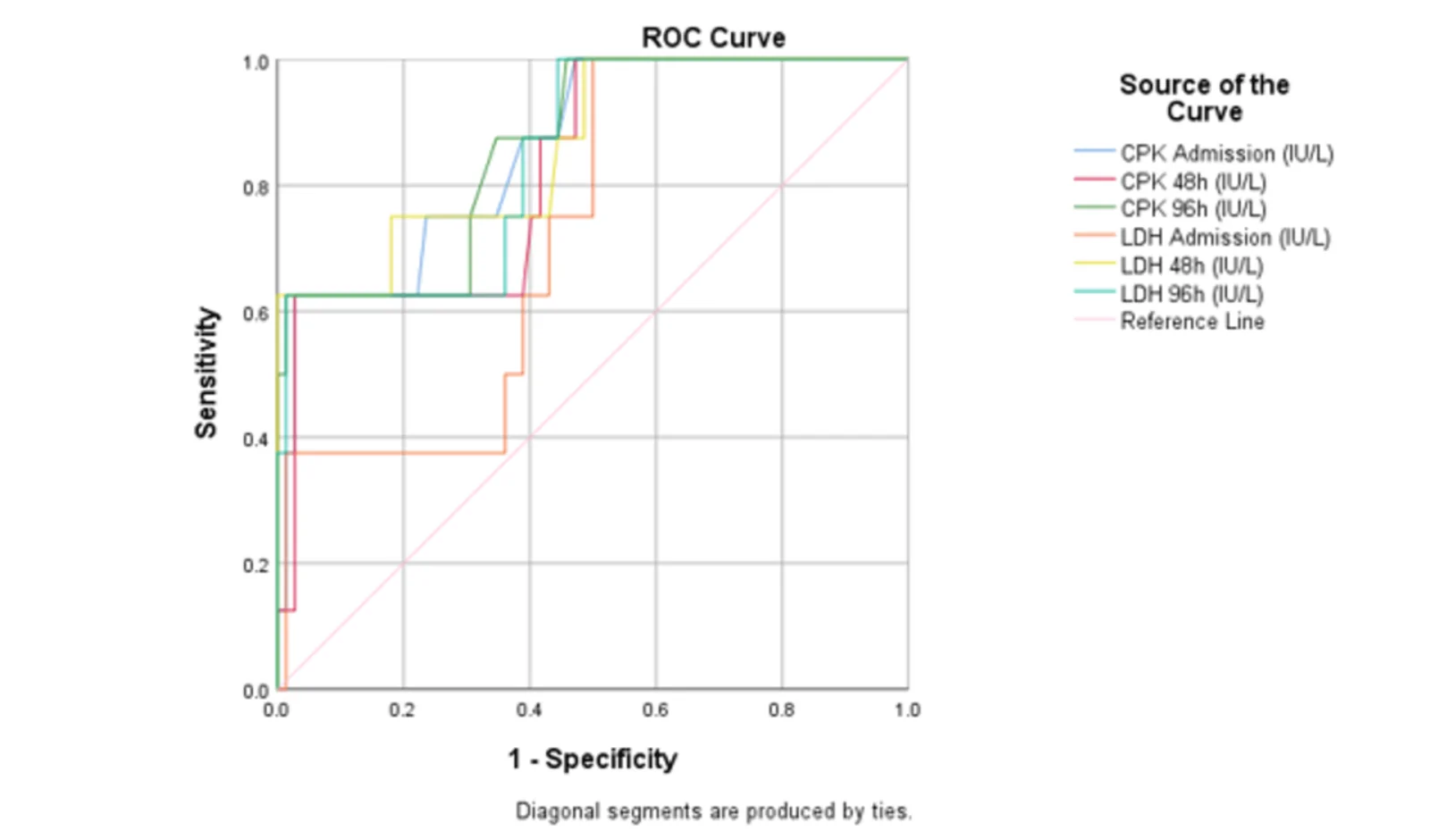
Receiver operating characteristic curves for intermediate syndrome. Curves are shown for serial CPK and LDH levels at admission, 48 hours, and 96 hours, using the development of intermediate syndrome as the positive actual state. Sensitivity is plotted against 1 − specificity. Discriminative performance is expressed as the area under the receiver operating characteristic curve (AUC), with corresponding 95% confidence intervals and p-values reported in Table 5. Statistical significance was defined as p < 0.05. CPK, creatine phosphokinase; LDH, lactate dehydrogenase; ROC, receiver operating characteristic; AUC, area under the curve; IMS, intermediate syndrome.

**Figure 5:**
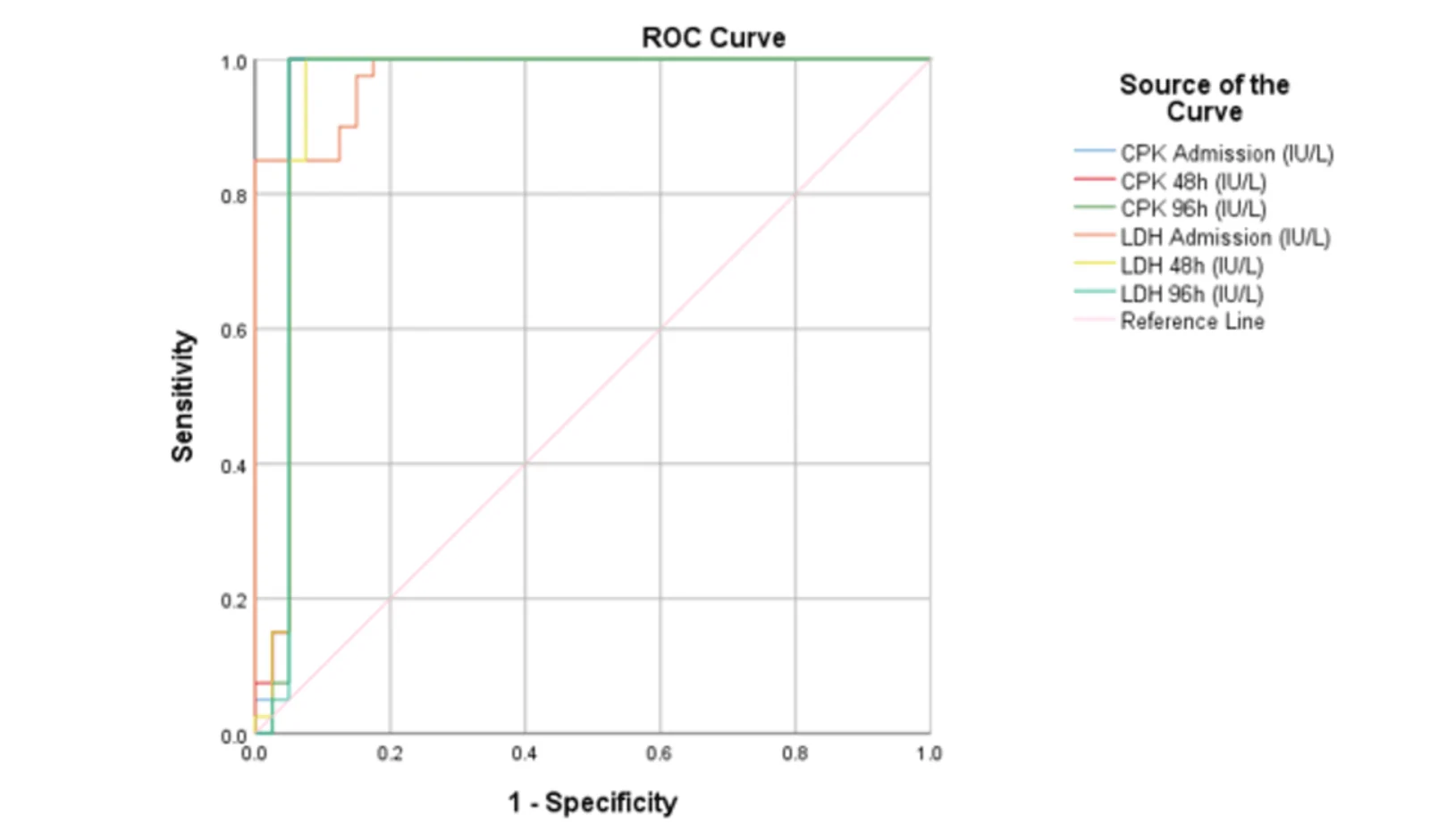
Receiver operating characteristic curves for moderate poisoning. Curves are shown for serial CPK and LDH levels at admission, 48 hours, and 96 hours, using moderate poisoning as the positive actual state. Sensitivity is plotted against 1 − specificity. Discriminative performance is expressed as the area under the receiver operating characteristic curve (AUC), with corresponding 95% confidence intervals and p-values reported in Table 5. Statistical significance was defined as p < 0.05. CPK, creatine phosphokinase; LDH, lactate dehydrogenase; ROC, receiver operating characteristic; AUC, area under the curve.

Clinical outcomes

Patients with moderate poisoning had a longer mean duration of hospitalization than patients with mild poisoning (11.42 ± 2.26 days vs. 7.58 ± 1.34 days). Patients who developed intermediate syndrome also had a longer hospital stay than those who did not develop intermediate syndrome (14.00 ± 1.31 days vs. 9.00 ± 2.30 days). Mortality occurred in three patients (3.8%), all of whom belonged to the moderate poisoning group. Mortality was numerically higher among patients with intermediate syndrome, with two of eight patients (25.0%) with intermediate syndrome dying compared with one of 72 patients (1.4%) without intermediate syndrome. As only three patients (3.8%) died, mortality-related biomarker findings were interpreted cautiously.

## Discussion

In this prospective observational study of 80 adults with mild-to-moderate organophosphorus compound poisoning, serial serum CPK and LDH levels were significantly associated with clinical severity and the development of intermediate syndrome. Both biomarkers showed a rise during the first 48 hours, followed by a decline at 96 hours, paralleling the clinically important period during which delayed neuromuscular complications may emerge. Patients with moderate poisoning and those who developed intermediate syndrome had higher biomarker levels throughout hospitalization.

Organophosphorus poisoning produces toxicity through acetylcholinesterase inhibition, leading to excessive stimulation of muscarinic, nicotinic, and central nervous system receptors [2,3]. Although acute cholinergic features often improve with atropinization and supportive therapy, intermediate syndrome may develop after the initial phase and can involve the respiratory muscles [4,5]. The biomarker pattern observed in this study supports the concept that muscle injury and systemic tissue damage may continue beyond the initial cholinergic phase, particularly among patients with more severe poisoning or subsequent intermediate syndrome.

The present findings are consistent with earlier studies. John et al. described muscle injury in organophosphorus poisoning and its relationship with intermediate syndrome [10]. Kumar et al. reported that serum CPK may help predict intermediate syndrome, with peak elevation during the early post-exposure period [11]. Bhattacharyya et al. reported that serum CPK correlated with clinical severity assessed by the POP scale [9]. In the present study, both CPK and LDH correlated positively with the severity score, suggesting that combined serial monitoring may provide useful adjunctive information.

An important strength of this study is the serial assessment of both CPK and LDH at admission, 48 hours, and 96 hours. This allowed evaluation of the temporal biomarker trajectory rather than relying on a single admission value. The rise in CPK and LDH at 48 hours is particularly clinically relevant because this time point falls within the typical 24-to-96-hour period during which intermediate syndrome develops. This temporal increase in muscle-associated biomarkers provides biological plausibility that evolving muscle injury may precede or accompany the clinical manifestations of intermediate syndrome. The finding that LDH at 48 hours and CPK at 96 hours showed high AUC values for intermediate syndrome further supports the potential role of serial biomarker assessment. Serial assessment, particularly at 48 hours, may therefore help identify patients who warrant closer clinical and respiratory monitoring, although prospective validation is required before these biomarkers can be used to guide therapeutic decisions.

ROC curve analysis showed excellent discrimination for moderate poisoning and good discrimination for intermediate syndrome. However, the absence of cut-off values, sensitivity, specificity, and predictive values limits direct bedside application. The mortality ROC findings should be considered exploratory because there were only three deaths, resulting in unstable estimates and a substantial risk of overestimation of the observed AUC values. Therefore, while elevated biomarker levels were associated with more severe illness and intermediate syndrome, the present data should not be interpreted as proving reliable mortality prediction.

In this cohort, accidental exposure predominated, accounting for 75% of cases. This differs from several studies in which intentional self-poisoning was more frequent. Accidental and intentional exposures may differ in the amount and circumstances of exposure and, consequently, in the severity of poisoning. The predominance of accidental exposure in our cohort may therefore have contributed to the relatively low mortality rate of 3.8% and may also have influenced the magnitude and temporal patterns of CPK and LDH elevation observed in this study. The low mortality may additionally have been influenced by the exclusion of patients with severe poisoning at presentation, early treatment, and standardized supportive management. Therefore, the observed biomarker trends and clinical outcomes should be interpreted in the context of the exposure pattern of this cohort, and their generalizability to populations predominantly comprising intentional self-poisoning may be limited. The longer duration of hospitalization among patients with moderate poisoning and intermediate syndrome further supports the clinical relevance of severity-based and biomarker-based risk stratification.

The findings suggest that serum CPK and LDH may be useful, inexpensive, and readily available adjuncts in the assessment of mild-to-moderate organophosphorus compound poisoning. These markers should be interpreted alongside clinical examination, severity scoring, respiratory monitoring, and response to atropine and oxime therapy. They should not replace clinical judgment, especially because intermediate syndrome is primarily a clinical diagnosis.

This study has several limitations. It was a single-center observational study with a modest sample size. Furthermore, the use of consecutive non-random sampling may have introduced selection bias and may limit the generalizability of the findings to other populations and healthcare settings. Only patients with mild-to-moderate poisoning were included; therefore, the findings may not be generalizable to patients with severe poisoning requiring immediate ventilatory support or profound neurological compromise at presentation. The severity classification was based on a modified POP-based score used in the study proforma, which may affect comparability with studies using the original unmodified scale. Serial cholinesterase levels were not evaluated alongside CPK and LDH, and long-term neurological follow-up was not performed. Only three deaths occurred, limiting mortality-related subgroup analysis and ROC interpretation.

Future studies with larger sample sizes, inclusion of severe poisoning cases, and serial cholinesterase measurements are needed to determine and externally validate clinically useful CPK and LDH cut-off values with corresponding sensitivity, specificity, and predictive values before these biomarkers can be recommended for bedside decision-making.

## Conclusions

In adults with mild-to-moderate organophosphorus compound poisoning, serial serum CPK and LDH levels were significantly associated with poisoning severity and development of intermediate syndrome. Both biomarkers showed an early rise during the first 48 hours, followed by a decline at 96 hours, with higher levels among patients with moderate poisoning and those who developed intermediate syndrome.

These inexpensive and readily available biomarkers may serve as useful adjuncts to clinical assessment for monitoring and risk stratification. However, they should not replace clinical evaluation or severity scoring, and their role in mortality prediction requires further study because only three deaths occurred in the present cohort.
